# A Magnetically Transformable Twisting Millirobot for Cargo Delivery at Low Reynolds Number

**DOI:** 10.1002/aisy.202401028

**Published:** 2025-05-19

**Authors:** Moonkwang Jeong, Jiyuan Tian, Meng Zhang, Tian Qiu

**Affiliations:** ^1^ Cyber Valley group ‐ Biomedical Microsystems Institute of Physical Chemistry University of Stuttgart Pfaffenwaldring 55 70569 Stuttgart Germany; ^2^ Division of Smart Technologies for Tumor Therapy German Cancer Research Center (DKFZ) Site Dresden Blasewitzer Str. 80 01307 Dresden Germany; ^3^ Faculty of Medicine Carl Gustav Carus Dresden University of Technology Dresden Germany; ^4^ Faculty of Electrical and Computer Engineering Dresden University of Technology Dresden Germany

**Keywords:** cargo delivery, flexible body, helical propeller, magnetic actuation, magnetic transformation, soft miniature robots

## Abstract

Inspired by bacteria flagella, miniature robots often use a helical shape to propel themselves in fluids at low Reynolds numbers. The helical microstructures in the robots are often rigid and are made by advanced 3D micro‐/nanofabrication techniques. However, it remains challenging to fabricate these 3D helical structures without complicated machinery. Herein, for the first time, a magnetically transformable millirobot—TwistBot—with a flexible body that can transform from a simple flat ribbon to a helical shape under an applied magnetic field is reported, enabling its propulsion in viscous fluids. The robot's twisting is modeled using numerical simulation and its geometry is optimized to maximize the twist angle. The unique shape transformation not only allows the propulsion through narrow lumens but also facilitates TwistBot in carrying and delivering solid cargo successfully to the target. The concept of the TwistBot opens new opportunities in designing soft transformable minirobots for targeted cargo delivery.

## Graphical abstract

Herein, a soft millirobot, TwistBot, that transforms from a flat shape to a helical shape to propel in fluids at low Reynolds number is reported. Its geometry is optimized by numerical simulation and validated by experiments to effectively propel and deliver solid cargos. The design paves the way for adaptive miniature robots for targeted cargo delivery in biological lumens.

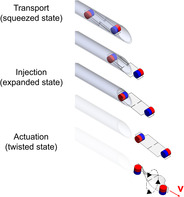

## 1 Introduction

In fluids at small scales, microorganisms experience viscous forces that dominate over inertial forces, which is known as the low Reynolds number regime (Re << 1). In this regime, the Navier–Stokes equation simplifies to the linear Stokes equation. The resultant flow is instantaneous and time‐reversible, and the flow parameters are dependent solely on time‐dependent boundary conditions with no other explicit time dependence.^[^ ^1^ ^]^

At low Re, a slender cylinder experiences asymmetric drag when moving along its main axis compared to when it moves perpendicular to the axis^[^ ^2^ ^]^ and moves at an angle of attack when pulled obliquely. Microorganisms, for example, *Escherichia coli* bacteria, follow this principle to rotate their helical flagella to generate effective propulsion in fluid environments.^[^ ^2^ ^]^ In nature, microorganisms exhibit adaptability by changing their morphologies and motion mechanisms in response to microenvironmental changes and functional demands.^[^ ^2^, ^3^, ^4^ ^]^ For example, the flagellar shape of the spermatozoa of *Echinus esculentus* changes to a helical shape in viscous environments, and much higher viscosity triggers the shape to become quasiplanar.^[^ ^5^ ^]^ The adaptability to fluidic properties changes is important for effective propulsion.

Inspired by nature, significant advances have been achieved in small‐scale robots to propel in fluids. Wireless actuation schemes for small‐scale robots vary widely, including magnetic,^[^ ^6^, ^7^, ^8^, ^9^, ^10^, ^11^, ^12^, ^13^, ^14^, ^15^, ^16^, ^17^ ^]^ acoustic,^[^ ^18^, ^19^, ^20^, ^21^ ^]^ and optical actuation.^[^ ^22^, ^23^, ^24^ ^]^ Magnetic fields are particularly utilized in medical applications due to their minimally invasive feature, high actuation force/torque, and precise controllability.^[^ ^25^, ^26^, ^27^ ^]^ Rotating a rigid helical structure by a rotating magnetic field is one of the most common ways for small‐scale robots to propel.^[^ ^1^, ^4^, ^25^, ^28^, ^29^, ^30^, ^31^ ^]^ For example, a magnetic screw in centimeter size actuated at low Reynolds number to drill through biological tissue,^[^ ^32^ ^]^ microscopic artificial bacterial flagella made by photolithography and self‐scrolling technique,^[^ ^33^ ^]^ helical nanopropellers made by glancing angle deposition,^[^ ^6^, ^34^ ^]^ and helical robots fabricated by two‐photon lithography.^[^ ^35^, ^36^, ^37^ ^]^ Similar to microorganisms that can adapt their shape and mechanisms according to environmental changes, advanced small‐scale robots have also been developed to transform the shape of helical tails, for example, a hydrogel‐based programmable robot that can transform due to changes in temperature and magnetic fields^[^ ^3^ ^]^ and a deformable helical tail made of responsive hydrogel to enhance its performance.^[^ ^7^, ^38^ ^]^ However, the helical shapes in the current robots are predefined during the fabrication process, which often requires complicated 3D microfabrication techniques, such as stress engineering and self‐scrolling, glancing angle deposition, or two‐photon lithography.^[^ ^28^ ^]^ Soft robots have recently demonstrated locomotion using various motion modalities. For instance, they can swim in fluids using helical‐shaped propellers and spirals,^[^ ^29^, ^31^, ^37^, ^39^ ^]^ while padel robots can crawl on surfaces.^[^ ^40^, ^41^, ^42^, ^43^ ^]^ To the best of our knowledge, it has not been shown that a planar microstructure can be reversibly twisted by a magnetic field into a helical shape to enable propulsion and cargo delivery at low Re.

In this article, we present the TwistBot, a magnetically driven millirobot that can transform its body shape in response to an external magnetic field for effective propulsion and cargo delivery at small scales in viscous fluids. The TwistBot features a flexible body that changes from a flat ribbon to a twisted helical shape under a homogeneous magnetic field. Upon the rotation of the magnetic field, the helical soft robot also rotates and propels through the fluids. It can effectively propel through viscous fluids with the wireless control of the propulsion direction. Three different body designs, characterized by varying numbers of slits, are also investigated to assess their performance. A solid cargo made of hydrogel is encapsulated by the flat‐helix shape transformation, and it is then released on the reverse process. The TwistBot concept introduces an adaptive shape soft robot that is easy to fabricate and effective to propel through fluids for targeted cargo delivery.

## 2 Results and Discussion

### 2.1 The TwistBot Design: A Magnetically Transformable Robot

**Figure 1:**
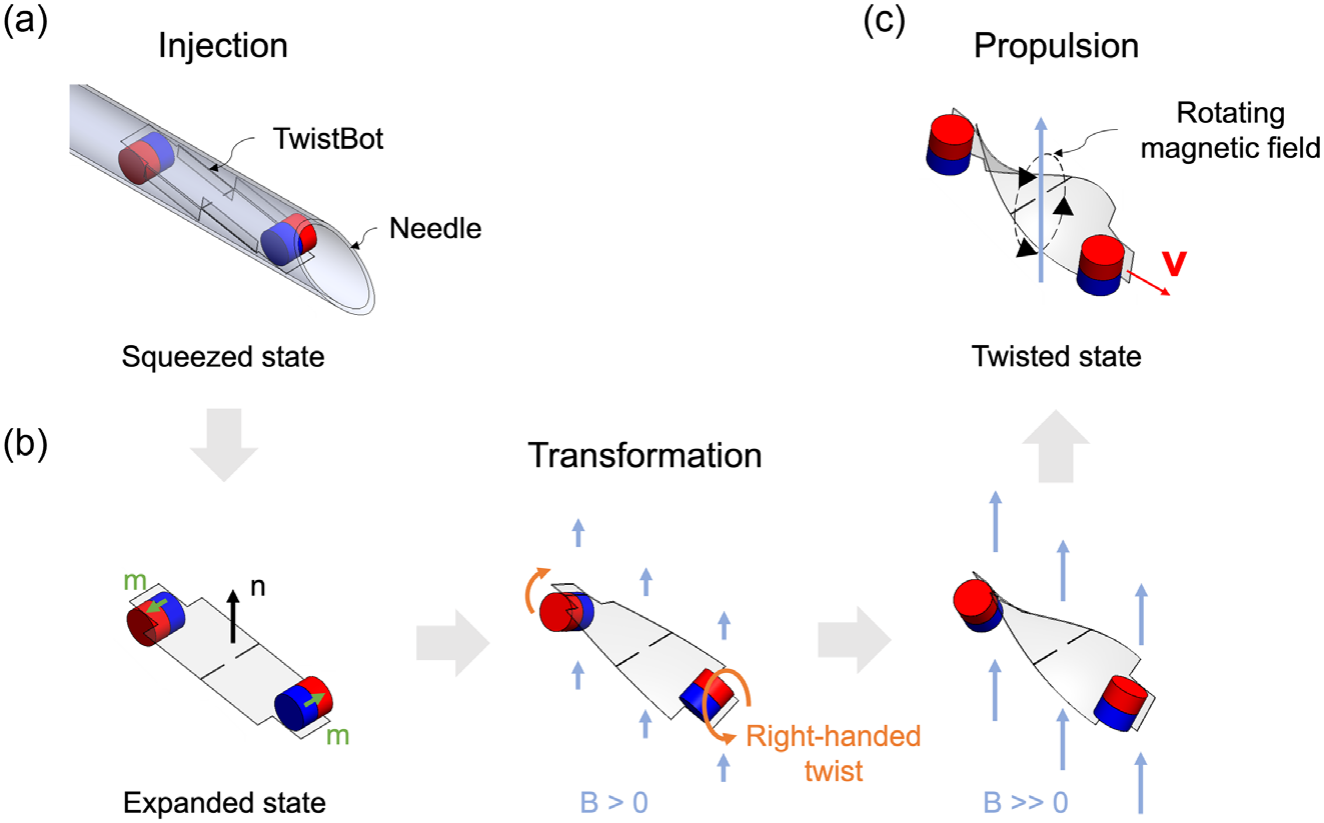
Schematic illustration of the shape transformation of the TwistBot. a) Injection: the TwistBot in the squeezed state to be transported and injected by a needle. b) Transformation: in a homogeneous external magnetic field, the TwistBot transforms its flexible body into a helical shape. The right‐handed shape is illustrated here, but a TwistBot may have both chiralities (details in Figure S1, Supporting Information). **m** indicates the magnetic moments at the end of the flexible body, and **n** indicates the normal vector of the flexible body. c) When the external field rotates, the twisted robot follows the rotation and propels with a linear velocity. B indicates the external homogeneous magnetic field shown by the blue arrows. The black arrow indicates the rotation directions of the magnetic field. The current approach utilizes laser cutting of standard PET films and commercially available permanent magnets, making it suitable for mass production. Moreover, the planar design supports scalability and enables potential miniaturization through thinner films and smaller magnets. For example, femtosecond laser cutting can be a promising method for fabricating the flexible body, while magnetic microparticles can be used as smaller magnetic actuators.

**Figure** **1** shows the process of TwistBot from injection to actuation and propulsion. The robot consists of a polymeric flat ribbon with two opposite permanent magnetic dipoles attached to both ends. Thanks to the flexible body, the robot exhibits a squeezed state for injection, which decreases its width up to 50% (Figure 1a). After injection, the robot is at the expanded state and is ready for shape transformation (Figure 1b). When a homogeneous magnetic field is applied, the ribbon is twisted to a helical shape, either left‐handed or right‐handed helix, as illustrated in Figure S1, Supporting Information. Interestingly, a TwistBot only has its chirality determined at the stage of transformation. The chirality is defined based on the angle β between the applied external magnetic field **B** and the normal vector **n** of the soft body at the planar state, where the normal vector **n** is defined using the curl right‐hand rule: the four fingers curl in the direction of the two magnetic moments (**m**) at each end of the soft body, and the thumb points in the direction of the normal vector **n**. When **B** is in or close to the opposite direction of **n** (90°<|β|≤180°), the robot twists to a left‐handed helix (Figure S1a–d, Supporting Information); vice versa, when **B** is in or close to the same direction of **n** (0°≤|β|<90°), the robot twists to a right‐handed helix (Figure S1e–h, Supporting Information). The twist angle is up to 180° until the two magnetic moments are aligned. As a result, the left‐ or right‐handed twisted robot propels in two different directions under the same direction rotational magnetic field due to translation–rotation coupling of the helical shape (Figure S1d or h, Supporting Information). This special feature of the transformable robot can be used to reprogram the propulsion direction on demand, which cannot be achieved by traditional rigid helical robots. The potential of the TwistBot also lies in the coordinated deployment of multiple robots for biomedical applications. The TwistBot has the with controlled chirality, which may enable unique propulsion behavior to achieve independent control strategy^[^ ^44^, ^45^ ^]^ of multiple TwistBots in future studies. In the experiments in this article, we used right‐handed TwistBots, i.e., the magnetic field **B** is applied in the same direction of the normal vector **n** at the initial state to transform the robot (Figure 1b).

### 2.2 The Magnetic Actuation Setup

**Figure 2:**
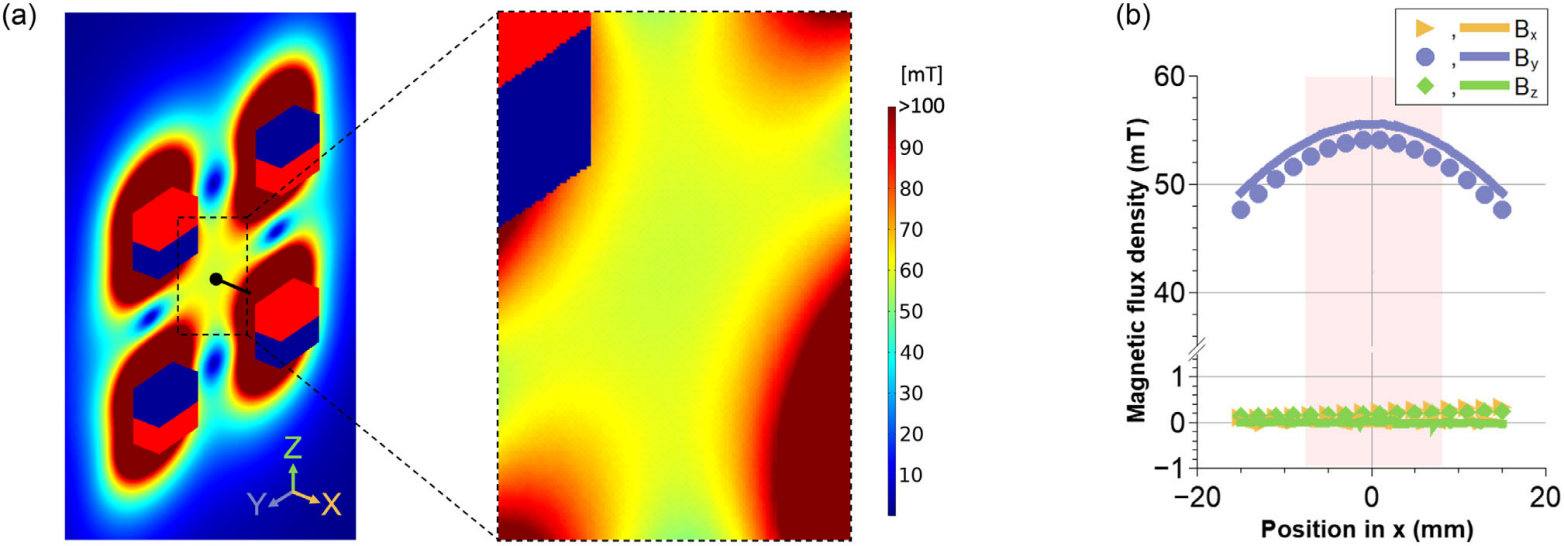
Characterization of the magnetic actuation setup. a) FEM simulation result. The center point (indicated as the black dot) is located at the center of the four magnets, and the center line (indicated as the black line) is parallel to the *x*‐axis and passing through the center point. The enlarged view of the working volume where a robot is actuated is shown on the right side. b) The superimposed magnetic flux density along the center line is experimentally measured and numerically simulated. Dots indicate experimental results, and solid lines represent FEM simulation results. The shaded regions highlight the working volume in b.

A TwistBot transforms its shape in response to an external magnetic field. Depending on the applied magnetic field, the actuators generate torque that twists the flexible body into a helical shape. When a robot is subjected to a rotating magnetic field, the helical‐shaped TwistBot follows the rotation around its helical axis and generates propulsive force. The magnetic field is realized by a customized magnetic actuation setup, modified based on our previous setup.^[^ ^46^ ^]^ The setup consists of four identical permanent magnets that are arranged in the shown directions and rotate in the same direction at the same frequency (details in Experimental Section). **Figure** **2**a and S2, Supporting Information, show the finite element method (FEM) simulation result. A relatively homogeneous magnetic field is generated in the center of the working volume of 15 × 15 × 15 mm^3^.

The magnetic flux density along the center line was measured experimentally and compared to the simulation results. As shown in Figure 2b, the maximum magnetic flux density appears at the center point (≈54 mT), and it decreases with distance from the center point. The difference between the measurement and the simulation is less than 3%. The measurements show good agreement with the simulation results. In this work, the working distance is limited to minimize the effect of magnetic gradient while securing the volume for actuation. The distance is around 15 mm (highlighted in pink), which has approximately ±3% difference in magnetic flux density in the moving direction (*x*‐axis).

### 2.3 Characterization of the Shape Transformation

**Figure 3:**
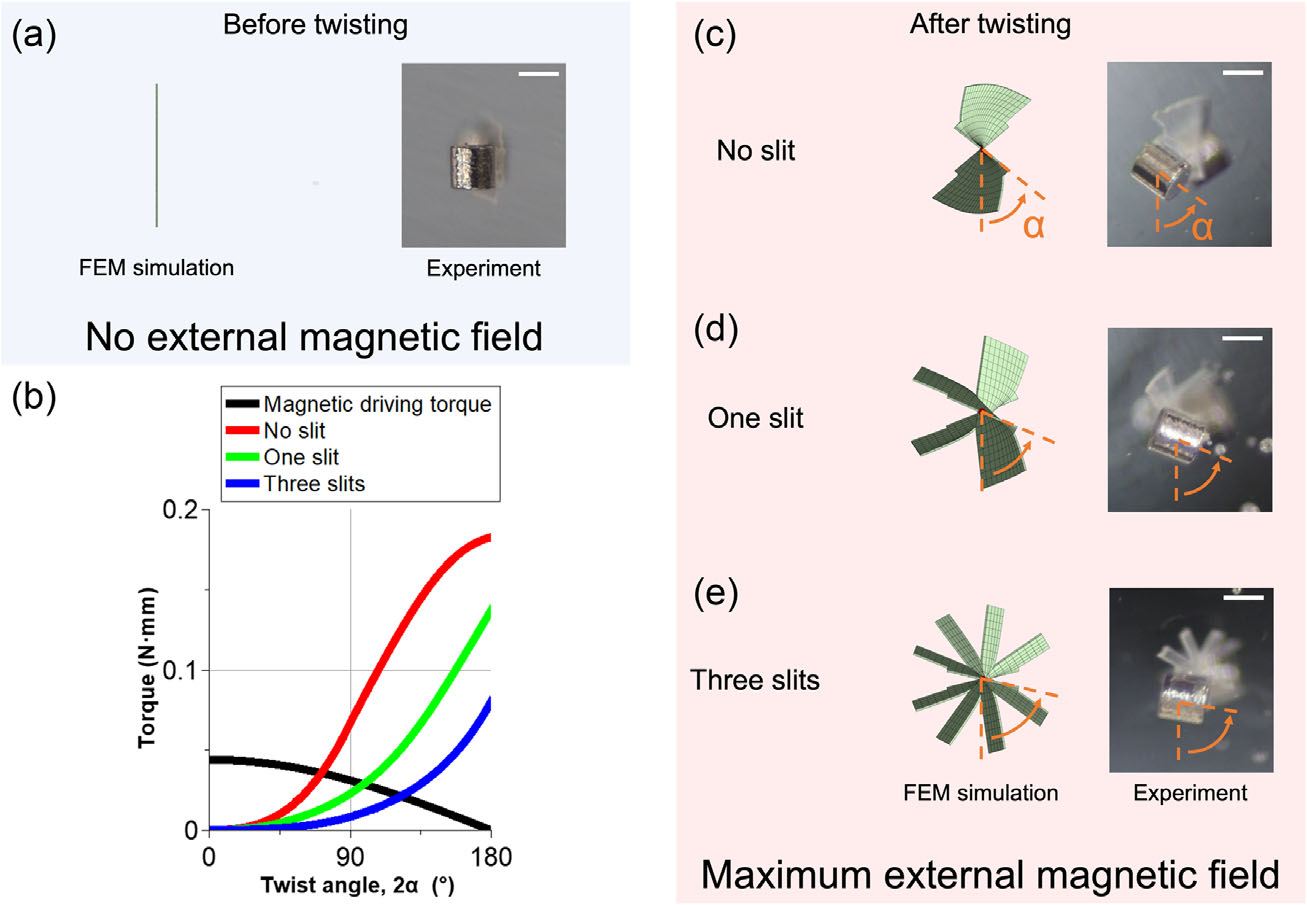
Characterization of the twist angles for different flexible body geometries. a) Before twisting with no external magnetic field. b) The elastic restoring torques (the three colorful curves) and the magnetic driving torque (the black curve) at various twist angles. The intersection points of the restoring torque and the driving torque determine the twist angle of the geometry, respectively. c–e) FEM simulation results (left column) and experimental results (right column) are in good match for the geometries of (c) no slit, (d) one slit, and (e) three slits. *α* is measured as the rotated angle of the front magnet relative to the external magnetic field in the vertical direction. All scale bars are 1 mm.

A TwistBot consists of two components: two actuators and one flexible body. The magnetic torque generated by the two actuators in the presence of an external magnetic field deforms the flexible body. When no magnetic field is applied, the robot has a flat shape, as shown in **Figure** **3**a (before twisting). However, when a sufficient magnetic field is applied, the actuators generate enough magnetic torque to twist the elastic body, as shown in Figure 3c–e (after twisting).

In the static state, the magnetic torque balances with the elastic restoring torque of the body, and the twist angle of the body is determined by its geometrical design. We designed the TwistBot with three geometries with different numbers of slits: no slit, one slit, and three slits. The flexible body is fabricated using a 23 μm thick polyethylene terephthalate (PET) film. The addition of slits on the body increases the twist angle, i.e., decreases the helical pitch to facilitate more effective propulsion in fluids, for a given magnetic torque. Each end of the body is coupled with two permanent magnets serving as actuators, with opposite directions of the magnet moments (see detailed dimensions in Figure S3, Supporting Information). The twist angle increases as the number of slits increases, which is due to the reduction in the torsional constant or the second moment of area of the cross section at the slits’ positions.

For a quantitative comparison, we conducted FEM simulation to calculate the restoring torques of three elastic bodies with continuous twist angles, as shown in red, green, and blue curves in Figure 3b. Their intersection points with the theoretical values of the magnetic driving torque (modeled analytically in Experimental Section) at different torsion angles (black curve in Figure 3b) determined the torsion angles of these three geometric models under magnetic fields, with each design achieving a twist angle of 72°, 99°, and 122°, respectively.

**Figure 4:**
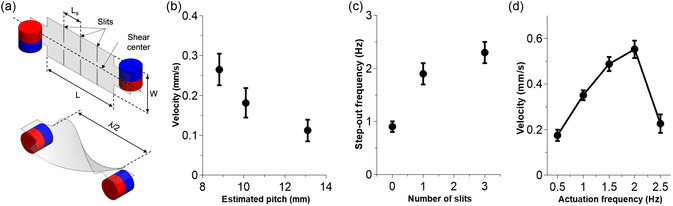
TwistBot Characterization. a) Schematic showing the key geometric parameters of the flexible body: length (L), width (W), and slit distance (L_s_) are defined before twisting, while pitch (λ) is measured after twisting. b) Average propulsion velocity over pitch at a fixed rotational frequency of 0.67 Hz. c) Step‐out frequency comparison among three designs with varying slit numbers. d) Average propulsion velocity of a TwistBot with three slits over input frequency.

The twisting deformations of these three models are shown in the left column of Figure 3c–e (FEM simulation results), while the corresponding experimental results, indicating the twist angle of each design at the maximum magnetic field (≈54 mT), are shown in the right column. In the FEM simulation, angular displacement (from 0° to 180°) was applied on one end (see details in Figure S4, Supporting Information), whereas both ends rotated during the experiment, defining the twist angle as 2*α*. The results show that an increased number of slits results in larger twist angles, with each design achieving an average twist angle (2*α*) of 111° ± 2°, 135° ± 2°, and 160° ± 3°, respectively (images from a different perspective are available in Figure S5, Supporting Information). The error is due to the assumptions of the FE model (detailed methods in Section 4). In particular, the FE model does not shorten the axial length during twisting. Introducing a nonlinear solver into the FE analysis will hopefully reduce the error, but this approach necessitates a trade‐off with computational cost. Based on the measured and simulated twist angles, the helical pitches of the flexible body for a given length of 4 mm are calculated and summarized in **Table** **1**. The twist angle increases with the addition of slits, which is consistent with the experimental results. The variation in helical pitch significantly affects the robot's propulsion performance (see Section 2.4 and **Figure** **4**).

**Table 1:** Summary of the twist angle (2*α*).

		No slit	One slit	Three slits
Twist angle [°]	Experiment	111 ± 2	135 ± 2	160 ± 3
	Simulation	72	99	122
Estimated pitch [mm]	Experiment	13.1	10.7	8.8
	Simulation	20	14.5	11.8

A benefit of the TwistBot is its transformable shape, which changes its pitch depending on the applied external magnetic field. Due to the geometrical factor, the maximum twist angle is limited to 180°. The torques required to reach the maximum twist angle are compared. For example, to achieve a twist angle of the maximum 180°, the body with three slits requires less than half of the driving torque (≈45%) compared with the body without any slits. It shows that an optimized geometry with slits dramatically decreases the requirement for the driving torque; therefore, it facilitates a smaller magnetic actuation setup or the miniaturization of the magnetic actuators.

### 2.4 Characterization of the Propulsion Performance

After twisting into a helical shape, the external field rotates to actuate the TwistBot's rotation and propel it through viscous fluids. Figure 4 illustrates the relationship between the number of slits, the helical pitch, and the TwistBot's velocity, as defined by the geometric parameters in Figure 4a. The pitch decreases from 13.1 mm with no slits to 8.8 mm with three slits, creating a tighter helix. Figure 4b shows the average propulsion velocity of a TwistBot in a viscous fluid at a rotational speed of 0.67 Hz. The velocity increases with the number of slits, reaching up to 0.26 mm s^−1^ with three slits from 0.11 mm s^−1^ with no slits.

The TwistBot can propel with stable motion up to a certain step‐out frequency.^[^ ^47^ ^]^ The step‐out frequency (Figure 4c), induced by the combined effect of increasing viscous force and the limitation in structural stability, increases with the number of slits from 0.9 Hz (no slit) to 1.9 Hz (one slit), and to 2.3 Hz (three slits). To compare the propulsion velocity of different designs, a fixed rotational speed of 0.67 Hz, which is lower than the lowest step‐out frequency, was used in the following experiments. With 0.67 Hz rotation, the maximum velocity of the three‐slit design is 0.26 mm s^−1^ for a robot with a diameter of 1.4 mm, and the calculated Re = 3.7 × 10^−6^ << 1. The higher number of slits results in a lower pitch of the helix and a faster propulsion speed. This is consistent with previous reports in the literature^[^ ^48^ ^]^ that in viscous fluids, with a given rotational speed, a smaller helical pitch leads to a faster propulsion. With the current designs, the effect of pitch is almost saturated as the angle already reaches 163° with the three‐slit design. Figure 4d shows the velocity curve of the TwistBot with three slits. The velocity is proportional to the frequency up to the step‐out frequency. The robot exhibits unstable motion near the step‐out frequency and tends to collapse as the frequency increases. In controlled environments with silicone oil, which offers a stable viscosity and minimizes external flow disturbances, the fastest speed reached by TwistBot is ≈0.6 mm s^−1^. This indicates that the robot should be able to propel against a fluidic flow that is lower than the fastest speed at low Reynolds number. Future work could explore the dynamic effects of TwistBot's propulsion in the flow of biofluids.

It is evident that a robot with a shorter pitch achieves a higher velocity. Two factors play a crucial role in twisting: the material stiffness and the cross‐sectional shape. Lowering the stiffness allows for larger twisting deformation. With a given material and a constant magnetic torque, lowering the torsional constant,^[^ ^49^ ^]^ i.e., the side length, by introducing the slits increases the twist angle, and thus the robot achieves a shorter pitch and achieves a faster propulsion. These factors, along with the applied magnetic torque, must be carefully balanced to optimize deformation and structural stability, ultimately enhancing propulsion performance.

### 2.5 Wireless Control of a TwistBot

**Figure 5:**
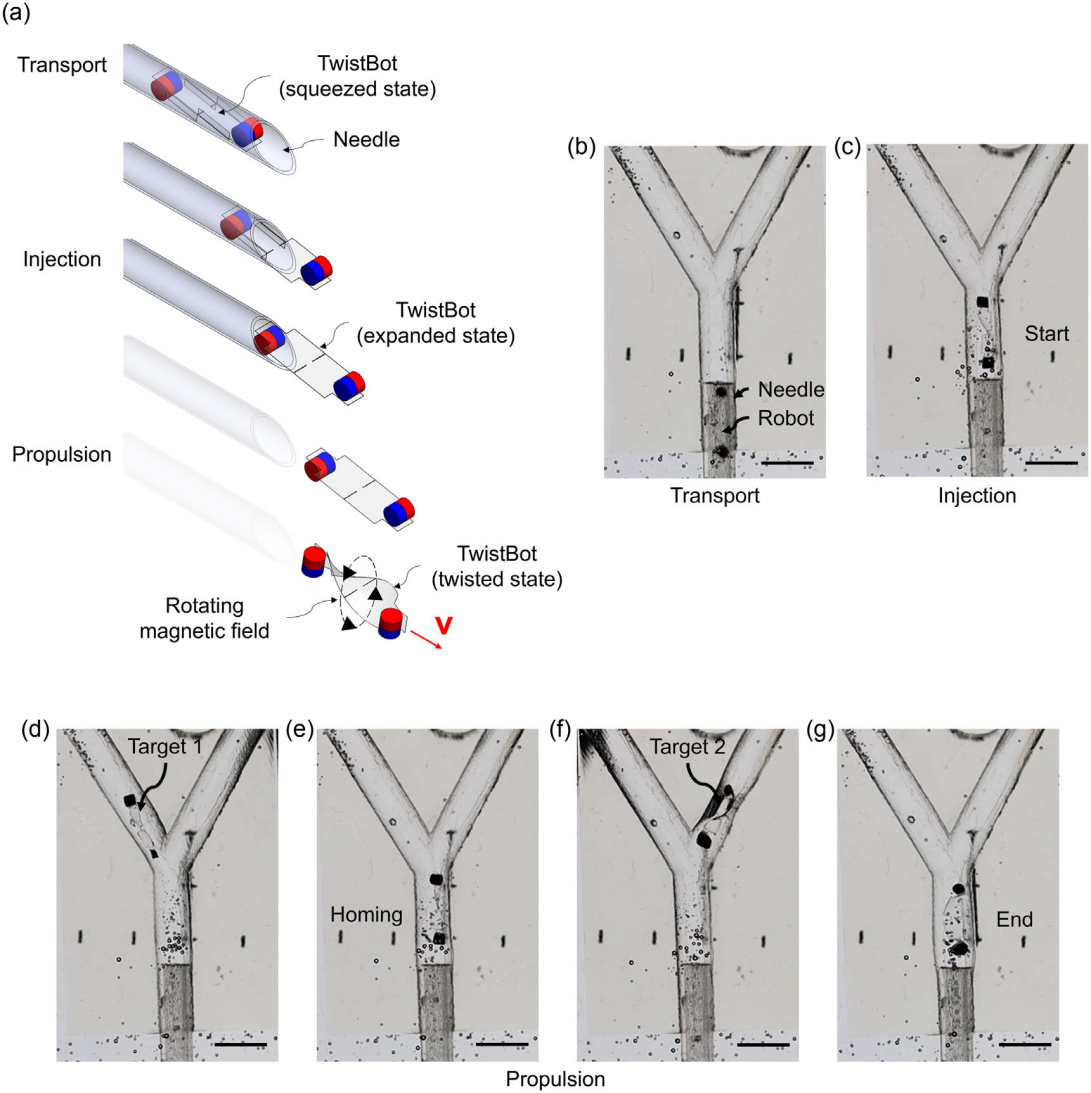
Control of the TwistBot in a Y‐shaped fluidic channel. a) Illustration of the transport of a TwistBot (in a squeezed state) in a needle, the injection (transformed to an expanded state), and the propulsion by a rotating magnetic field (in a twisted state). b–c) Snapshots of the injection of the TwistBot by a 3D‐printed needle. d–g) Controlled propulsion of the TwistBot to two target locations in different branches. All scale bars are 5 mm.

The magnetic actuation setup can control the rotation direction of the magnets, which drives the TwistBot to propel forward or backward (along the centerline labeled in Figure 2a). Additionally, the steering of the rotational axis of the magnetic field is achieved in plane by rotating the actuation setup relative to the TwistBot. The propulsion direction of the TwistBot tends to align with the rotational axis and can therefore be controlled in‐plane, as shown in **Figure** **5**. Moreover, the flexible body is soft enough to squeeze into a narrow channel of a needle to minimize the injection footprint. Figure 5a shows the process of transport, injection, and propulsion. After injection, a TwistBot in a squeezed state expands its flexible body into an expanded state. Once the injection is complete, the TwistBot twists its body in response to an external magnetic field and actuates in a viscous fluid driven by a rotating magnetic field.

Video S1 demonstrates robot injection and the controlled propulsion process. First, the TwistBot is transported by a 3D‐printed needle to inject it into a Y‐shaped fluidic channel (Figure 5b). After the robot is released into the channel, a homogeneous magnetic field is applied to twist the robot, and then it starts to rotate to actuate the robot (Figure 5c). The rotational magnetic field axis is steered to the left with an angle of 35° to reach the target 1 (Figure 5d). When it reaches the target, the magnetic field rotates in the opposite direction to reverse the robot's propulsion back to the home position (Figure 5e). Similarly, the robot is steered to a second target in the right branch (Figure 5f). This result demonstrates the in‐plane control capabilities and robustness of the TwistBot. The robot occasionally collides with the channel wall during steering, but its structure with two magnetic actuators stays stable. The robustness of the structure is enhanced by the magnetic interaction with the strong, homogeneous magnetic field. With further mechanical enhancements to the magnetic actuation setup, this design approach can be extended to facilitate 3D navigation within biological lumens in future applications.

### 2.6 Solid Cargo Delivery by the TwistBot

**Figure 6:**
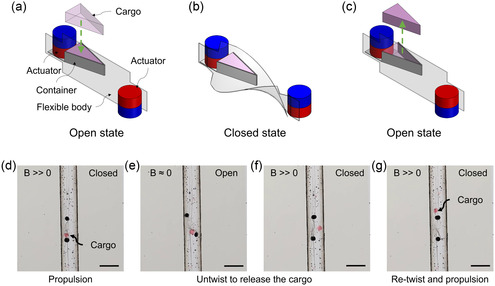
Cargo delivery by a TwistBot with the transformable body shape. Schematic illustrations of a) the open state for loading the cargo, b) the closed state with cargo locked in the container during propulsion, and c) the open state for releasing the cargo. d) The robot propels while carrying the cargo (a hydrogel block with red dye) in its container in the closed state by its twisted body. e) Decreasing the magnetic field induces the untwisting of the robot and thus the opening of the container for cargo release. f,g) With the magnetic field, the robot is twisted again and propels away to leave the cargo. All scale bars are 5 mm.

The TwistBot transforms from a flat shape to a twisted shape, which offers additional possibilities to open and close an on‐board container to carry and release solid cargos (**Figure** **6**). The container is designed to be a triangular shape to fit the twisted helical shape and is attached to the top of one actuator. One side of the container has an opening to allow the loading and unloading of the cargo (Figure 6a). The opening is blocked by the twisted robot body (Figure 6b) to hold the cargo in place during propulsion. When the robot reaches the target, the magnetic field is turned off, and the robot returns to the flat shape, which clears the opening of the container to release the cargo (Figure 6c). Figure 6d–f shows the sequential snapshots of the cargo transportation and delivery process with a TwistBot (see also Video S2, Supporting Information). Upon reaching the designated location, the magnetic actuation setup is moved away from the robot to reduce the magnetic field's intensity. This action allows the robot to untwist and open the container. The robot is then manipulated using a rotating magnetic field with a lower field strength, inducing oscillatory motion that helps the cargo to be released. Another critical factor influencing the release performance is the interaction between the cargo and the container. The size and the shape of the cargo are carefully designed to fit the cargo chamber (see Figure S3i, Supporting Information), ensuring low resistance during the release process. After cargo release, the magnetic field is applied again to retwist the robot, and it propels in the opposite direction to leave the target location for retrieval.

## 3 Conclusion

In this article, we report a TwistBot that can transform from a flat shape to a helical shape in order to propel in fluids at low Reynolds number. The special feature allows the robot to be injected by a thin needle. The optimization of the robot's geometry is achieved by FEM simulation and validated by experiments. The robots show the capability of in‐plane propulsion, as well as carrying solid cargo and controlled release at the targeted location owing to the reversible shape transformation. The TwistBot can be applied to propel in various viscous fluids within the human body, including pathological mucus with elevated viscosity in pulmonary diseases. While healthy airway mucus has a low viscosity (≈0.01 Pa·s),^[^ ^50^ ^]^ mucus in conditions such as cystic fibrosis and chronic obstructive pulmonary disease significantly increases mucus viscosity (≈110 Pa·s).^[^ ^51^ ^]^ Silicone oil (≈97 Pa·s) was used for experimental validation due to its controllable viscosity and uniformity. Future studies will explore the robot's performance in physiologically relevant biofluids. Therefore, the TwistBot design may lead to adaptable and easy‐to‐fabricate miniature robots for targeted cargo delivery in biological lumens.

## 4 Experimental Section

### Fabrication of the TwistBot

A TwistBot comprises two components: two actuators and one elastic body. The actuator is a cylindrical permanent magnet magnetized along the longitudinal direction (NdFeB‐N45, Ø 1 mm, 1 mm in height, Supermagnete, Germany). The flexible body, made from PET film (23 μm thick, Mylar, 785‐0782, RS Components GmbH, Germany), was designed in SOLIDWORKS 2021 (Dassault Systemes, France) and fabricated using a laser cutting machine (BEAMBOX PRO, FLUX Europe, Belgium). The flexible bodies in different designs are shown in Figure S3, Supporting Information, captured using a stereoscope (OZP 552, KERN & SOHN GmbH, Germany) and a camera (a2A2448‐75ucPRO, Basler AG, Germany). The magnets were assembled using an adhesive (Loctite 401, Henkel, Germany) on the opposite sides of the flexible body. For cargo delivery experiment, a container was fabricated using the laser cutting machine (BEAMBOX PRO) and attached to the top of an actuator using adhesive (Loctite 401). The body and container designs are shown in Figure S3, Supporting Information.

### Magnetic Actuation Setup

The TwistBot was actuated using a customized magnetic actuation setup.^[^ ^46^ ^]^ The actuation setup was built based on four sets of permanent magnets. Each set was assembled using two identical magnets (NdFeB‐N45, 30 × 30 × 15 mm^3^, Supermagnete). The center‐to‐center distance between sets of magnets is 95 mm. The four sets of magnets were connected to a stepper motor (SM2862‐5251, Sanyo Denki, Japan) with a timing belt mechanism. The magnets rotated synchronously under the control of a microcontroller (Arduino UNO, Italy). The magnetic flux density was scanned by a gaussmeter (HGM09s, MAGSYS magnet systeme GmbH, Germany) using a motorized translational stage (LTS300C/M, Thorlabs GmbH, Bergkirchen, Germany). The magnetic flux density was verified by numerical simulation in COMSOL Multiphysics using the AC/DC Module (Magnetic Fields, No Currents, v5.6, COMSOL AB, Stockholm, Sweden). The median remanence specified in the datasheets of the magnets,^[^ ^52^ ^]^ i.e., 1.345 T for N45, was used for simulation. The feature called Magnetic Flux Conservation was used as the boundary condition to consider only the magnetic field originated by the permanent magnets. The boundary was defined as air (200 × 200 × 200 mm^3^). The distance between the TwistBot and the magnetic actuation setup was controlled to vary the magnetic flux density for the transformation of the flexible body using a motorized translational stage (LTS300C/M, Thorlabs GmbH). The steering of the robot was achieved by a motorized rotational stage (HDR50/M, Thorlabs GmbH).

### Characterization of the TwistBot

The TwistBot was tested in silicone oil (100,000 cSt, 378437, Sigma–Aldrich, Germany) to evaluate its performance. The test of each kind of TwistBot was repeated at least five times. The locomotion of the TwistBot was recorded using a camera (EOS RP, Canon, Japan) equipped with a 60 mm lens (Canon). The recorded videos were analyzed using ImageJ (version 1.53 t, National Institutes of Health, USA) for motion analysis. To calculate the velocity of each design, we measured the distance travelled and the time taken. We then computed the average and the standard deviation based on the experiments. Outliers, defined as the maximum and minimum values in each group, were excluded from the analysis. The Y‐channel and the needle were 3D‐printed using a photopolymer (VeroUltra ClearS, Stratasys, Minnesota, United States) on a 3D printer (J35 Pro, Stratasys). The Y‐channel has a diameter of 3 mm, while the needle has an inner diameter of 1.7 mm (Figure 5). First, a TwistBot in a squeezed state was injected into the Y‐channel using the 3D‐printed needle connected to a syringe. The syringe and the Y‐channel were filled with silicone oil (100,000 cSt, Sigma–Aldrich). Then, the robot was controlled by the rotating magnetic field to navigate the target locations. For the cargo delivery experiment, a straight channel with a diameter of 3 mm was 3D‐printed using a photopolymer (VeroUltra ClearS, Stratasys) on the J35 Pro (Stratasys) (Figure 6). The cargo was made of gelatin from porcine skin (3.4%wt, G1890, Sigma–Aldrich, Germany) mixed with reddish food dye (Natural food colors, Biovegan GmbH, Germany).

### FEM Simulation of the Twist Angle

Numerical analysis was conducted using ANSYS WORKBENCH (ANSYS, Inc., Canonsburg, PA, USA), in which the static structural module was applied. To ensure the convergence of the model mesh, the mesh size was refined from 0.5 to 0.1 mm and finally determined to be a hexahedral mesh (8‐node solid element: SOLID185) with a length of 0.2 mm and a width of 0.1 mm. Each simulation took about 40 mins. Large deformation was allowed during the simulation process. At the same time, the mesh was set to be axially symmetric in the whole structure to ensure the reliability of the results. In the FE model, the geometric and material assumptions are used as follows. Young's modulus of the PET film is 2164.8 MPa.^[^ ^53^ ^]^ The center of rotation of the robot in the FE model is along the axis of the film itself. Since the magnet and the film are bonded by strong glue, the bonding position of the magnet affects the length of the film. In the FE model, however, it is assumed that the magnet is located in the middle of the two structures, so the deformable length of the whole robot remains at 5 mm. The total length does not change while the body twists. The boundary conditions are shown in Figure S4, Supporting Information. The fixed constraint and angular displacement (from 0° to 180°) are shown on the left and right side of the model. Each boundary is located at a distance of 0.5 mm from the edge.

### Magnetic Driving Torque

Analytical solution of the magnetic driving torque is given as *τ* = *mB*sin*θ* where *τ* is the magnetic torque, *m* is the magnetic dipole moment, *B* is the magnetic field, and *θ* is the angle between the magnetic moment and the magnetic field. The magnetic dipole moment was calculated by *m* = *MV*. Here, *M* is the magnetization per unit volume, and *V* is the volume of the magnet. The magnetization is calculated using *M* = *B*_r_/*μ*_0_, where *B*_r_ is the remanence and *μ*_0_ is the vacuum permeability. The median remanence values (*B*_r_ = 1.345 T for N45) specified in the datasheets of the magnets.^[^ ^52^ ^]^

## Conflict of Interest

The authors declare no conflict of interest.

## Author Contributions

**Moonkwang Jeong**: investigation (lead); methodology (lead); validation (lead); visualization (lead); writing—original draft (lead); writing—review and editing (equal). **Jiyuan Tian**: investigation (supporting); writing—original draft (supporting). **Meng Zhang**: investigation (supporting); validation (supporting). **Tian Qiu**: funding acquisition (lead); investigation (supporting); supervision (lead); writing—review and editing (equal).

## Supporting information

Supplementary Material

## Data Availability

The data that support the findings of this study are available in the supplementary material of this article.
