# Supplementary material for: A Magnetically Transformable Twisting Millirobot for Cargo Delivery at Low Reynolds Number
Source: Adv Intell Syst. 2025 May 19;7(8):2401028. doi: 10.1002/aisy.202401028 (PMC12370169; doi:10.1002/aisy.202401028)
Supplement: Supplementary file 1 — Supplementary Material [file AISY-7-0-s001.zip › Aisy.202401028-sup-0001-suppdata-S1/TwistBot_Supporting_Information_Production_Data.docx]

Supporting Information

A magnetically transformable twisting milli-robot for cargo delivery at low Reynolds number

Moonkwang Jeong, Jiyuan Tian, Meng Zhang and Tian Qiu*


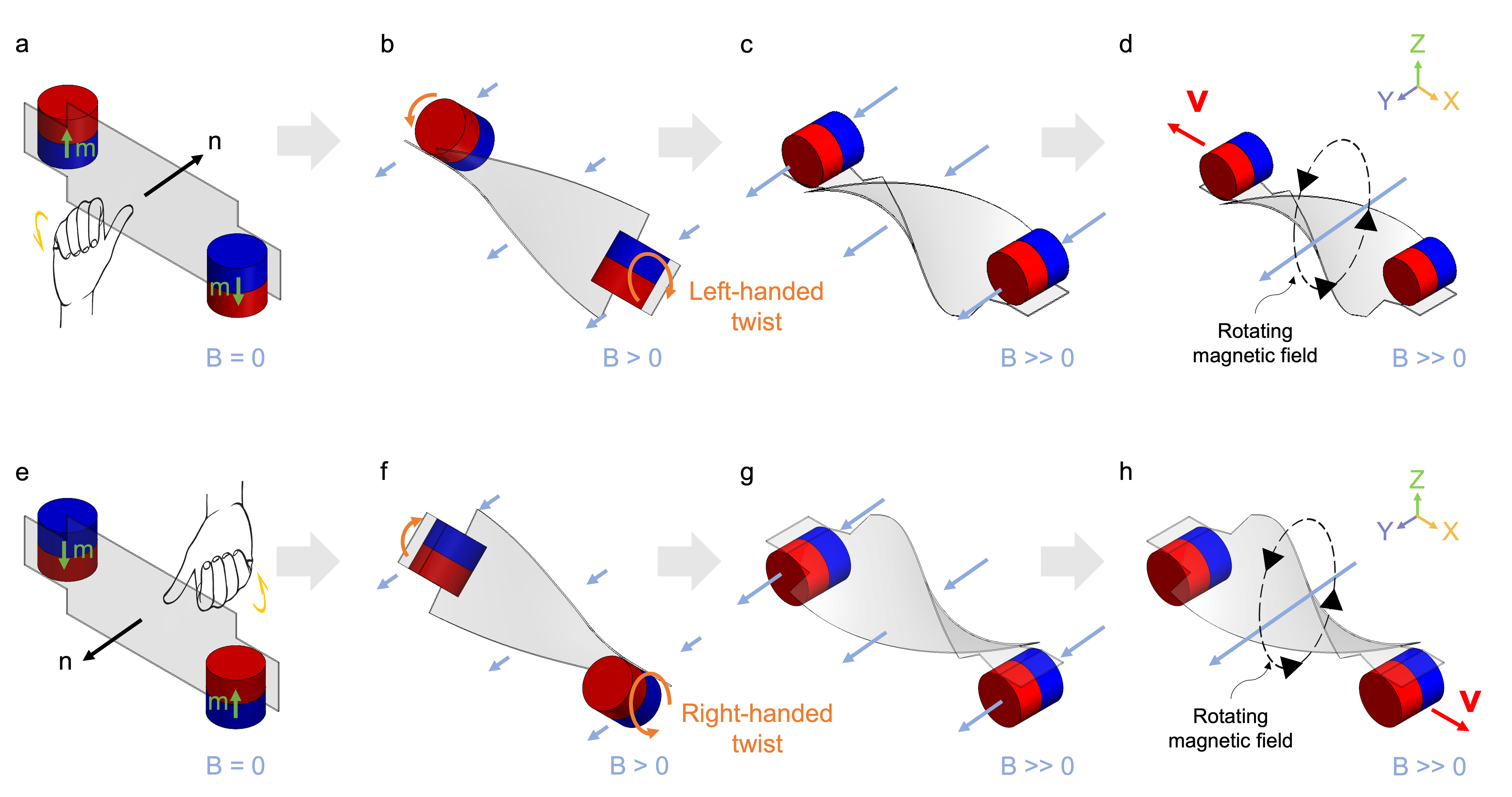


**Figure S1.** Schematic illustration of the TwistBot at different magnetic flux densities. (a and e) Initial states of a TwistBot without a magnetic field. **m** indicates the two magnetic moments at the end of the flexible body. Following the right-hand rule, we define the normal vector **n** of the flexible body. When the applied homogeneous external magnetic field **B** (blue arrows) is in the opposite direction of **n**, the robot is twisted to a left-handed helix (b-d); vice versa, when the applied field **B** is in the same direction of **n**, the robot is twisted to a right-handed helix (f-h). Depending on the chirality, a robot propels in the negative or positive x-direction (the linear velocity **V** is indicated by the red arrow) under the same direction rotational magnetic field (indicated by the black arrows).


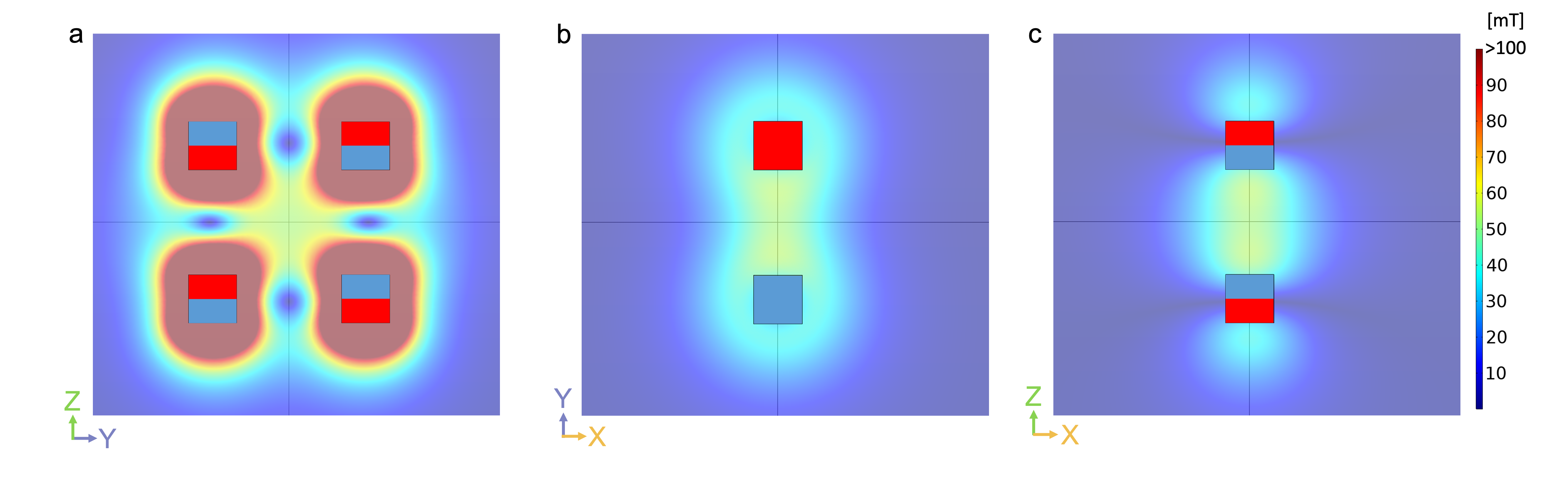


**Figure S2.** Numerical simulation results of the magnetic actuation set-up. Magnetic flux density is plotted in the (a) YZ-plane, x = 0, (b) XY-plane, z = 0 and (c) XZ-plane, y = 0.


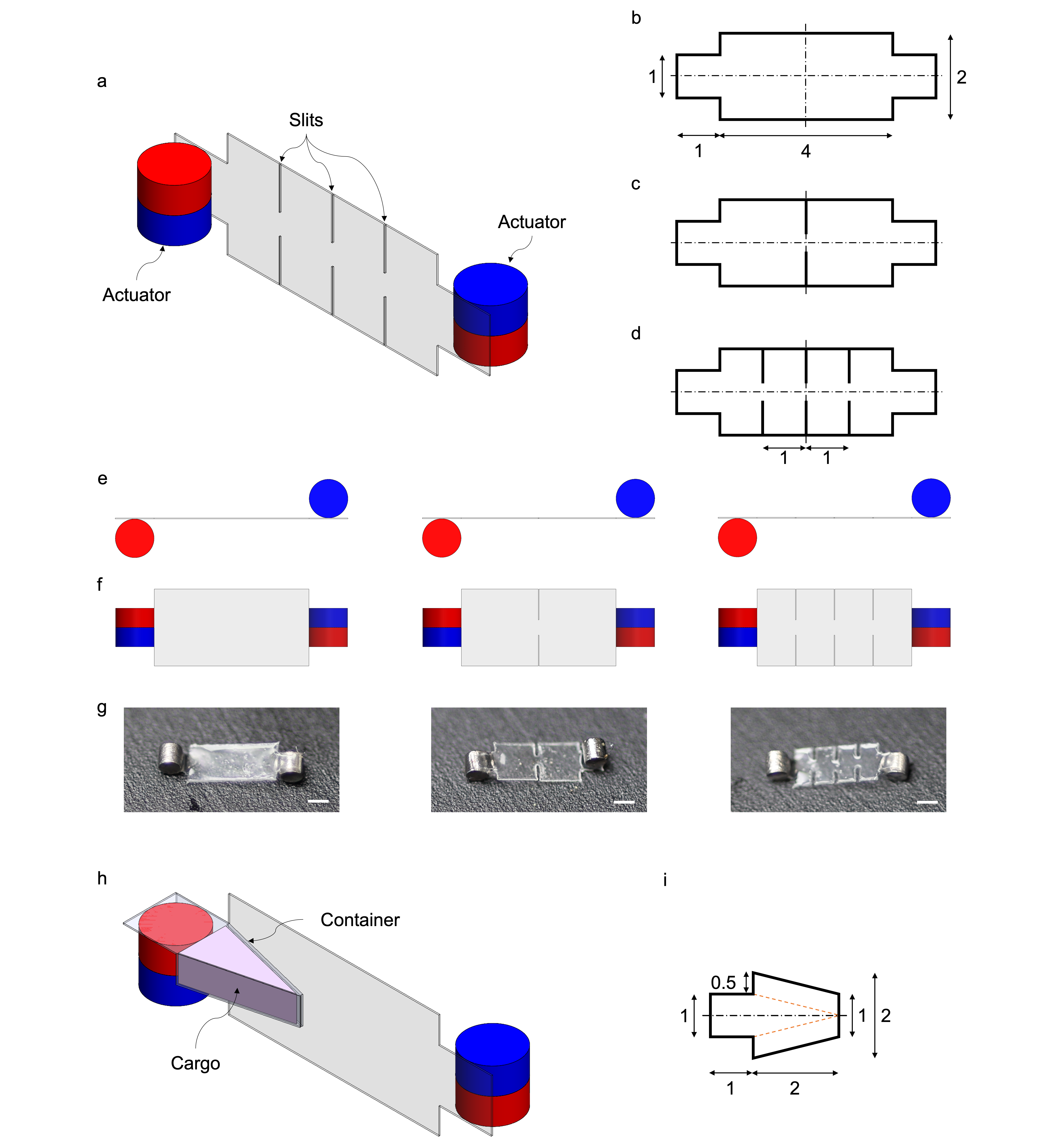


**Figure S3.** Detailed geometrical parameters of the TwistBot. (a) A schematic of a TwistBot that comprises two actuators and one flexible body with three slits. The mechanical drawings of the flexible body with (b) no slit, (c) one slit, and (d) three slits. Three designs of the robot with different slit numbers in the (e) top- and (f) side-view. (g) Images of the assembled TwistBots. All scale bars are 1 mm. (h) A schematic of a TwistBot with a cargo container and (i) The mechanical drawing of the container (the dashed lines indicate the folding lines). All numbers are in mm.


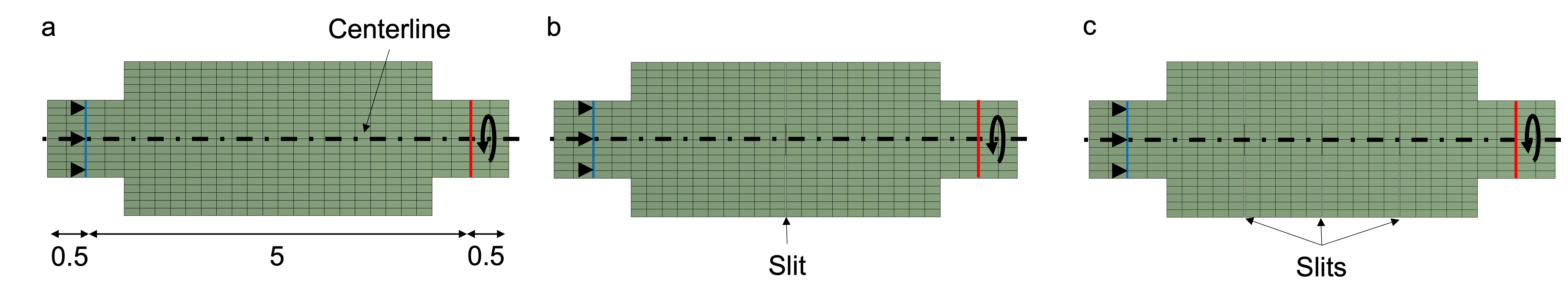


**Figure S4.** The numerical model and the boundary conditions for FEM simulation of the designs with (a) no slit, (b) one slit, and (c) three slits. All models have the fixed boundary on the left side (blue line), and the rotation is applied on the right side (red line) around the center line.


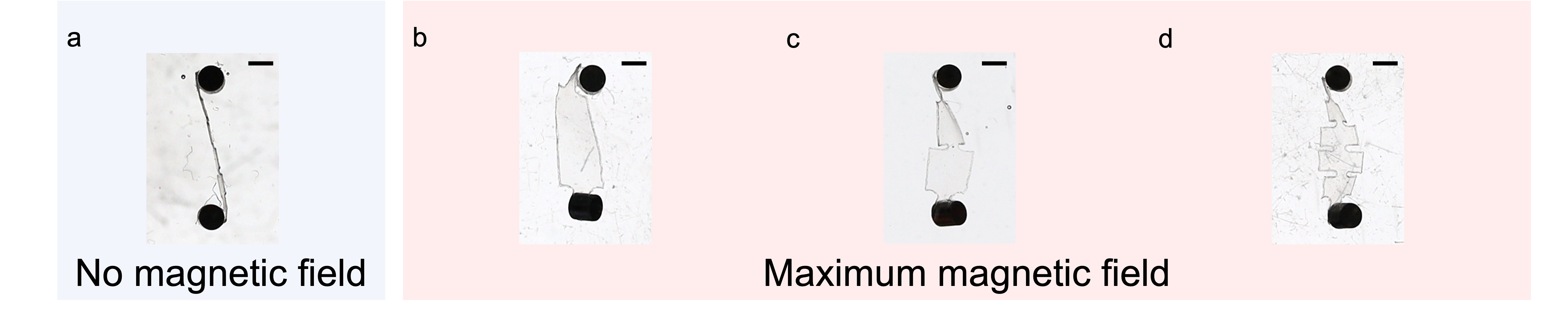


**Figure S5.** Images of the robots showing the twist angle from top view. (a-b) no slit, (c) one slit, and (d) three slits at (a) no magnetic field and (b-d) the maximum magnetic field B = 54 mT.
